# Machine Learning Models Utilizing Oxidative Stress Biomarkers for Breast Cancer Prediction: Efficacy and Limitations in Sentinel Lymph Node Metastasis Detection

**DOI:** 10.3390/biomedicines13123107

**Published:** 2025-12-17

**Authors:** José Manuel Martínez-Ramírez, Cristina Cueto-Ureña, María Jesús Ramírez-Expósito, José Manuel Martínez-Martos

**Affiliations:** 1 Department of Computer Science, University of Jaén, E-23071 Jaén, Spain; 2 Experimental and Clinical Physiopathology Research Group, CVI-1039, Department of Health Sciences, University of Jaén, E-23071 Jaén, Spain; ccueto@ujaen.es (C.C.-U.); mramirez@ujaen.es (M.J.R.-E.); jmmartos@ujaen.es (J.M.M.-M.)

**Keywords:** breast cancer, machine learning, oxidative stress, circulating biomarkers, metastasis, early detection, Random Forest, sentinel lymph node

## Abstract

**Objective:** This study aimed to apply the Random Forest machine learning model using oxidative stress biomarkers to classify breast cancer status and assess sentinel lymph node (SLN) metastasis, a pathology of high incidence and mortality that represents a major public health challenge. **Methods:** The breast cancer classification cohort included 188 women with infiltrating ductal carcinoma and 78 healthy volunteers. For SLN metastasis assessment, a subset of 29 women with metastases and 57 controls (n = 86) was used. Data preprocessing and the SMOTE technique were applied to balance the classes in the metastasis set, achieving a perfect balance of 171 examples (57 per class). Random Forest model with a leave-one-out validation strategy was employed and oxidative stress biomarkers (e.g., lipid peroxidation, total antioxidant capacity, superoxide dismutase, catalase, glutathione peroxidase) were used. **Results:** The model achieved high accuracy (0.996) in classifying breast cancer, representing a substantial improvement over current screening methods such as mammography. In contrast, its performance in detecting SLN metastases was more limited (accuracy = 0.854), likely reflecting the inherent complexity and heterogeneity of the metastatic process. Moreover, these estimates derive from a retrospective case–control cohort and should not be viewed as a substitute for, or a direct comparison with, population-based mammography screening, which would require dedicated prospective validation. **Conclusions:** The findings underscore the model’s robust performance in distinguishing women with breast cancer from healthy volunteers, but highlight significant gaps in its ability to diagnose metastatic disease. Future research should integrate additional biomarkers, longitudinal data, and explainable artificial intelligence (XAI) methods to improve clinical interpretability and accuracy in metastasis prediction, moving towards precision medicine.

## 1. Introduction

Breast cancer remains one of the most prevalent malignancies among women worldwide and represent a major public health challenge due to its high incidence and notable mortality rates [1]. The disease exhibits a complex epidemiologic profile that is influenced by a range of factors, including genetic predisposition, hormonal milieu, reproductive history, lifestyle behaviors, and environmental exposures. Although advances in screening and treatment have contributed to a gradual decline in mortality, incidence rates continue to rise, driven in part by risk factors such as early menarche, late menopause, nulliparity, obesity, and alcohol consumption [2,3,4,5,6,7]. According to the most recent global estimates, breast cancer incidence increased by 1% annually from 2012 to 2021, with a steeper increase among women younger than 50 years (1.4% per year) compared to those 50 or older (0.7% per year). Projections indicate that the global burden of breast cancer will continue to rise in the coming decades, with a higher impact on low- and middle-income countries where access to early detection and treatment remains limited [5,8].

Early detection remains strongly associated with better prognoses and a wider range of therapeutic options. Routine screening methods, particularly mammography, have been instrumental in shifting the distribution of stage at diagnosis toward more treatable, early-stage disease. Despite these advances, controversies remain regarding optimal screening intervals, the risk of overdiagnosis, and the psychological burden of false-positive results. In fact, recent studies have intensified debates regarding optimal screening protocols, particularly concerning overdiagnosis in older women. Overdiagnosis rates are substantial for women in their 70s and older, with rates of 31% for women aged 70–74 years, escalating to 47% for those 75–84 years, and reaching 51% for women over 85 years. These findings have stimulated ongoing research into refining screening protocols and incorporating individual risk stratification to tailor early detection efforts more precisely. Despite these challenges, approximately 66% of breast cancer cases are diagnosed at a localized stage when treatments tend to work better [9,10,11,12,13].

In the same way, predicting breast cancer metastasis remains a challenge. Metastasis is a multistep process involving local invasion, intravasation, survival in the circulation, extravasation, and colonization at distant sites, with each step influenced by an intricate network of genetic, epigenetic, and microenvironmental factors [14,15]. Recent advances in machine learning have shown promise in this area, with studies reporting predictive accuracies of up to 96% for identifying patients at risk of metastatic progression using genomic biomarkers. While gene expression profiling and molecular signatures have provided valuable insights into the metastatic potential of tumors, the variability observed among different patient cohorts and even within a single tumor over time continues to impede the development of applicable predictive models [6,16,17,18,19,20,21,22,23,24].

Lymph nodes are part of the immune system where they act, among other functions, as filters that help eliminate unwanted elements from the body. During the process of metastasis, lymphatic metastasis is the first step in the spread of tumor cells through the lymphatic vessels, leading to colonization of the lymph nodes. This process is regulated at multiple levels and is not a completely passive phenomenon, involving various molecular signals and cellular changes to facilitate the entry, colonization and survival of tumor cells in the lymph node [14,15]. Thus, the sentinel lymph node (SLN) is considered to be the first lymph node or group of lymph nodes to which cancer cells are most likely to spread from a primary tumor [25,26,27].

SLN biopsy allows, by means of a minimally invasive procedure, accurate axillary staging in order to proceed or not to proceed to complete axillary lymph node dissection. The status of these nodes is of great prognostic importance for breast cancer patients, as complete axillary dissection is associated with significant morbidity, including risk of upper limb lymphedema, paresthesia, pain and restriction of shoulder movement. By reflecting the status of the remaining lymph nodes in the drainage basin, a negative SLN result allows patients to avoid a complete axillary dissection. Once removed, the sentinel node is analyzed, often using the one-step nucleic acid amplification (OSNA) method. This method detects cytokeratin 19 (CK19) mRNA amplification and allows classification of nodes into negative (<250 copies/mL of CK19 mRNA), presence of isolated tumor cells (>100 but <250 copies/mL), presence of micrometastases (>250 but <5000 copies/mL) or presence of macrometastases (>5000 copies/mL). SLN with macrometastases or micrometastases may lead to the decision to perform a complete axillary dissection, while the presence of isolated tumor cells or a true negative result would avoid this additional surgery. As at the tumor level, the involvement of redox processes in metastatic progression has also been demonstrated at the SLN level [25].

Advances in imaging and molecular diagnostics have further reinforced the critical role of early detection in breast cancer management. Traditional imaging methods have been enhanced by improvements in resolution and algorithmic analysis, which allow for better visualization of tumors in dense breast tissue. Concurrently, research into circulating biomarkers offers promise for detecting malignancies at a subclinical stage. Among these biomarkers, those related to oxidative stress have received considerable attention. Circulating oxidative stress biomarkers have garnered significant attention as critical indicators of the redox state and overall oxidative balance in the human body. These biomarkers reflect the dynamic interplay between the generation of reactive oxygen species and the effectiveness of antioxidant defenses and have been extensively investigated in relation to numerous pathological conditions [28,29,30]. Oxidative damage is known to perturb cellular homeostasis, and its evaluation through serum biomarkers offers valuable insights into the mechanisms underlying various diseases, ranging from metabolic disorders to cancer. Among these biomarkers, lipid peroxidation a protein carbonylation products, total antioxidant capacity, and key antioxidant molecules and enzymes such as glutathione, catalase, glutathione peroxidase, and superoxide dismutase provide complementary perspectives on oxidative stress levels in vivo [24,31,32,33,34,35,36,37,38].

These developments are complemented by emerging computational methods, including artificial intelligence and machine learning, which aim to increase diagnostic accuracy and reduce inter-observer variability [39]. The integration of these innovations into established screening programs is paving the way toward a more personalized approach to early detection that could optimize patient outcomes by minimizing invasive procedures and enabling earlier intervention [4,6,7,9,40,41] and holds the potential to further reduce mortality while improving quality of life for patients [1,2,3,5].

Machine learning techniques have emerged as powerful tools in the field of cancer prediction, facilitating early diagnosis, prognosis, and personalized treatment strategies [39]. These computational methods build predictive models that can distinguish between benign and malignant conditions with increasing accuracy [40,41,42,43,44]. Although deep neural networks, particularly convolutional neural networks, have been applied successfully to radiological and histopathological imaging biomarkers, they have also been employed to integrate molecular profiles and clinical variables to generate comprehensive prognostic models. Recent studies have demonstrated that AI models can successfully predict breast cancer distant metastasis using clinical blood markers and ultrasound data, with combined models showing superior discriminatory ability and strong generalization performance [45,46,47,48,49,50,51,52,53]. Despite their impact cancer diagnostics, their inherent opacity persists as a critical limitation. Deep neural networks often operate as “black-box” systems, obscuring the specific parameters and features underpinning their predictions. This lack of interpretability poses significant challenges in clinical settings, where understanding the rationale behind diagnostic decisions is essential to underscore the imperative for prioritizing interpretability alongside performance in clinical AI deployment [21]. In contrast, explainable AI (XAI) models, such as interpretable linear classifiers or rule-based systems, achieve comparable diagnostic accuracy while providing explicit, human-readable representations of the variables and decision thresholds involved [53,54]. This approach enhances the precision of therapeutic interventions and contributes to the goal of reducing cancer-related morbidity and mortality by facilitating early and accurate intervention [40,44,55,56,57,58,59,60,61], including personalized oncology [45,62,63,64,65].

Our objective is to develop machine learning models that harness oxidative stress biomarkers to classify the presence of breast cancer and to evaluate their efficacy in detecting SLN metastatic disease in a retrospective cohort. Breast cancer remains a leading cause of cancer-related mortality largely due to metastasis, emphasizing the need for early and accurate identification of patients at high risk of developing metastatic lesions. Oxidative stress, which arises from an imbalance between the production of reactive oxygen species and the body’s antioxidant defenses, plays a crucial role throughout breast cancer development, from early tumorigenesis through metastatic progression, with ROS-induced DNA damage contributing to malignant transformation and treatment resistance [25,31,34,66,67].

Biomarkers used include lipid peroxidation and protein carbonylation products, total antioxidant capacity, non-enzyme antioxidants such as glutathione, uric acid and direct bilirubin, and the activities of superoxide dismutase, catalase and glutathione peroxidase, which reflect biochemical pathways perturbed in malignancy [25,31,34,36,37,38,66,67]. The incorporation of these biomarkers into machine learning algorithms could create classification models that can capture the subtle biochemical alterations associated with tumor aggressiveness and metastatic potential. This approach is grounded in prior evidence suggesting that oxidative stress not only contributes to carcinogenesis but may also serve as a quantifiable surrogate for the complex interplay between tumor biology and the host microenvironment, particularly as precision medicine continues to evolve toward more personalized treatment strategies [34,35,60,64,68,69,70].

## 2. Materials and Methods

### 2.1. Subjects

The study cohort comprised 188 women diagnosed with ductal infiltrating carcinoma at the University Hospital of Jaén’s Breast Pathology Unit and 78 healthy volunteers were used to build a model for breast cancer classification. Healthy volunteers were women with no personal history of breast cancer or other malignancies and with an unremarkable clinical evaluation at the time of blood sampling. Out of these, a subset containing exclusively those women with SLN metastasis (a total of 29 women), together with 57 patients without SLN involvement, was additionally extracted to analyze SLN metastasis status. This study was approved by the Ethical Committee of the University Hospital of Jaén and all subjects signed a term of free, informed consent. Patients were categorized into distinct clinical groups based on treatment protocols as previously described [25,66,71]. All procedures were in accordance with the ethical standards of the institutional and/or national research committee and with the 1964 Helsinki declaration and its later amendments or comparable ethical standards.

### 2.2. Datasets and Pre-Processing

This dataset includes data previously described in [25,66,71] and additional unpublished results related to the SLN status (presence of macrometastasis, micrometastasis and isolated tumor cells). The dataset includes biomarkers of oxidative stress (lipid peroxidation, protein oxidation and total antioxidant capacity), non-enzyme antioxidant defense systems (total glutathione, uric acid and direct bilirubin) and enzyme antioxidant defense systems activities (superoxide dismutase, catalase and glutathione peroxidase). All biomarkers were measured in serum using spectrophotometric and enzymatic assays as previously reported in these studies.

The dataset presented several limitations that required specific preprocessing steps before model training. A number of variables described clinical or treatment-related characteristics that only existed in women with breast cancer and had no counterpart in the control group. To avoid introducing an artificial separation between classes driven solely by these cancer-specific fields, all such variables were excluded from the feature set, while retaining every subject in the cohort because the records contained no errors and all cases were considered informative for the analysis. Beyond discarding these non-comparable attributes, no additional filtering of instances was performed.

**Figure 1:**
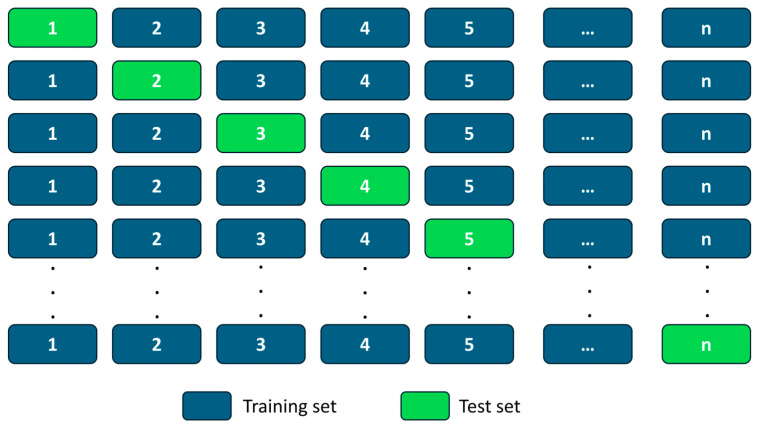
Illustration of the leave-one-out validation scheme, where each iteration trains the model on all but a single subject and then evaluates performance on that held-out case. The procedure cycles through the entire cohort so that every individual serves once as the test instance.

Lastly, the strategy used for the training of the models was a leave-one-out strategy, in which each model is trained with all examples of the dataset but one, which is used to test its accuracy. This process is repeated a number of times equal to the amount of examples on the dataset, so that every individual example can be used as test once, as seen in Figure 1. In practical terms, each woman is left out once, the model is trained on all the remaining subjects, and then it is asked to classify the left-out case.

### 2.3. Classification

Several models have been tested in the literature in an attempt to diagnose breast cancer. Particularly, deep learning models are the most common, oftentimes making use of images that are analyzed using models such as convolutional neural networks (CNN). Other approaches make use of machine learning methods in an attempt to either extract information from biomarkers or from images. Here, the Random Forest model was used to extract relevant information from biomarkers in order to classify breast cancer status (cancer vs. control) and, in a second model, SLN metastasis category in a given patient. The Random Forest model [72] is an ensemble model, and as such, it combines several simpler tree-based models, such as C4.5, to compute a final result. Conceptually, each tree corresponds to a set of simple “if–then” rules applied to the biomarker values, and the final decision is obtained by a majority vote across all trees [73].

What differentiates Random Forest from other ensemble algorithms is the fact that, for every tree, instead of the full set of attributes, only a fraction of them is considered. Once all trees have produced their outputs, their predictions are aggregated, using either weighted contributions or a simple majority vote, to assign a class label to each subject. Because every tree is built from a different subset of variables, combining them in this way yields a classifier that is more stable and less sensitive to the idiosyncrasies of any single tree, which helps to reduce overfitting in settings with many predictors.

In this study, the ensemble was implemented as a Random Forest constructed from C4.5 decision trees with a confidence factor of 0.25 and a minimum of two instances per terminal node. During tree induction, split points were not fixed to the original observed values but were optimized by selecting the thresholds that maximized classification performance [74]. The split criterion was the information gain ratio. Lastly, during the pruning phase, subtree raising was considered as to not fully remove them from the resulting tree. Furthermore, the model was tested by using several different numbers of trees.

In a C4.5 decision tree, internal nodes split the data into branches according to threshold values of specific predictor variables. The terminal, or leaf, nodes do not branch further but instead assign a class label, so that classifying a new case simply involves traversing the tree from the root to a leaf by comparing its attribute values with the learned split conditions.

The tree is constructed through several iterations of the same algorithm, developed in three phases. In the first phase, when all or almost all elements within a node belong to the same class or label, that node is considered a leaf and the procedure stops for it. In the second phase, if this condition is not met, all attributes of each element in the node are examined, and the attribute that provides the greatest information gain is selected; the node is then divided into several child nodes defined by threshold values of that attribute, allowing the elements of the original node to be distributed among them. In the third phase, once the tree has been built by repeating the previous two phases, pruning [75] may be applied to remove certain nodes in order to prevent overfitting and, in some cases, improve the accuracy of the model.

## 3. Results

The following sections present the results provided by both generated models. In Section 3.1, the results for the first model, which classifies breast cancer status using oxidative stress biomarkers, are shown. Furthermore, in Section 3.2, the results for the second model, which classifies SLN metastases using the same biomarkers, are provided. Overall results are shown in Table 1.

### 3.1. Breast Cancer Detection

The Random Forest model was able to achieve high results when classifying breast cancer using circulating oxidative stress biomarkers and the leave-one-out test–training split to ensure that results were not biased or subject to overfitting. By using 6 trees, the accuracy was 0.996, which remained stable when further increasing the number of trees. The confusion matrix can be found in Table 2. In this table, raw counts are shown for each true class and predicted class combination; corresponding row-wise percentages can also be included to facilitate interpretation of false-positive and false-negative rates.

**Figure 2:**
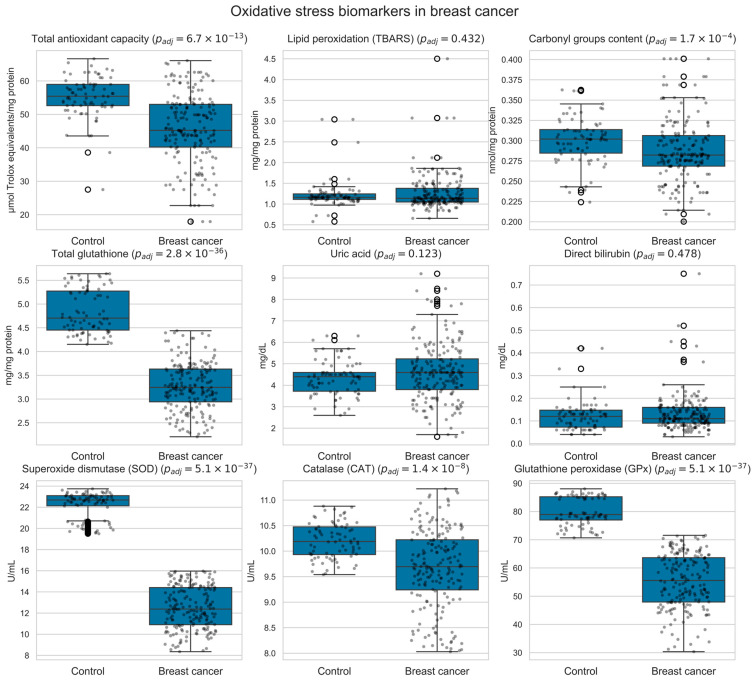
Box-and-whisker plots showing the distribution of circulating oxidative-stress biomarkers in women with breast cancer and healthy volunteers. Each panel displays a single biomarker, with overlaid jittered individual data points (black dots). *p*-values were obtained using two-sided Mann–Whitney U tests and adjusted for multiple comparisons using the Benjamini–Hochberg procedure; the adjusted *p*-value for each biomarker is indicated in the corresponding panel title. White open circles mark boxplot outliers.

Figure 2 summarizes the distribution of each oxidative stress biomarker in breast cancer patients and healthy controls.

The specific rules that each tree generated are provided in detail in Table 3, Table 4, Table 5, Table 6, Table 7 and Table 8.

### 3.2. SLN Metastasis

Additional pre-processing was applied for this task, as the dataset was both quite unbalanced and quite limited, including roughly three times as many control examples as metastasis-affected patients. Due to this, SMOTE [74] was used as an oversampling technique on the minority class. Several experiments were considered, using SMOTE to increase minority classes by 50%, 100%, 200% and to perfectly balance all classes, so that there were as many examples of each individual class. This last configuration offered the best results, allowing the dataset to achieve a total of 171 examples, 57 for each specific class that is to be classified (macrometastasis, micrometastasis and negative).

The Random Forest model has some limitations when using oxidative stress biomarkers to classify SLN metastases. While a leave-one-out test–training split was applied to reduce bias and avoid overfitting, and multiple numbers of trees were tested, the best results were achieved by using 50 trees, while the final achieved accuracy was of 0.854, proving that metastasis detection is a more complex task. The confusion matrix can be found in Table 9. As with the breast cancer model, raw counts and row-wise percentages can be shown to better illustrate the distribution of correct and incorrect classifications across SLN categories.

**Figure 3:**
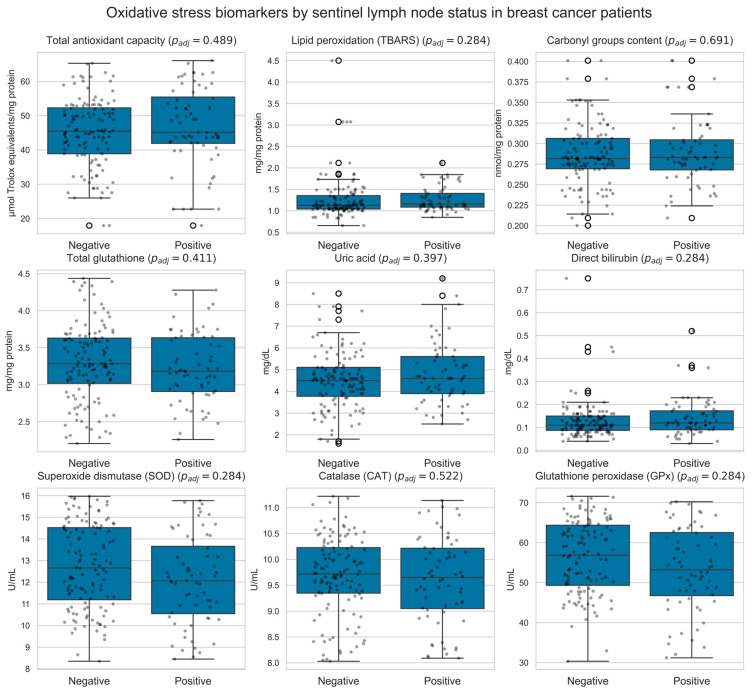
Box-and-whisker plots showing the distribution of circulating oxidative-stress biomarkers in women with breast cancer stratified by sentinel lymph node status (negative vs. positive). Analyses were restricted to patients in the cancer group. Each panel displays a single biomarker, with overlaid jittered individual data points (black dots). *p*-values were obtained using two-sided Mann–Whitney U tests comparing node-negative and node-positive patients and adjusted for multiple comparisons using the Benjamini–Hochberg procedure; the adjusted *p*-value for each biomarker is indicated in the corresponding panel title. White open circles mark boxplot outliers.

In addition, Figure 3 depicts the distribution of oxidative stress biomarkers among women with breast cancer stratified by sentinel lymph node status (negative vs. positive).

## 4. Discussion

Our study was designed to harness machine learning techniques by integrating oxidative stress biomarkers to classify the presence of breast cancer and to assess SLN metastasis status in a retrospective cohort, a significant global health challenge for women [1,8]. This work follows a cross-sectional case–control design, comparing women with a confirmed diagnosis of breast cancer and healthy volunteers at the time of sampling, rather than a prospective cohort with longitudinal follow-up. Our results indicate that the model can detect subtle biochemical alterations associated with tumorigenesis, thereby offering a promising tool for biomarker-based cancer classification. This work adds to other advances in machine learning applications for breast cancer detection, which have reported up to 98% accuracy in utilizing biochemical biomarkers for the distinction between breast cancer and control subjects; our model demonstrates a further increase in accuracy [76]. This finding is particularly relevant considering the limitations of existing screening methods, such as mammography, which may not be as effective in women with dense breast tissue or may lead to overdiagnosis and unnecessary anxiety [9]. In fact, mammograms miss cancer in dense breasts in almost half of such cases, with overdiagnosis rates reaching 31% for women aged 70–74 years and escalating to 51% for women over 85 years [77]. The incorporation of oxidative stress biomarkers into a machine learning framework offers a complementary approach to early detection, potentially improving the overall accuracy and reducing the burden associated with conventional screening methods [78,79].

Importantly, our design is that of a cross-sectional case–control study comparing women with a confirmed diagnosis of breast cancer and healthy volunteers, rather than a longitudinal cohort designed to estimate future incidence. As such, the model evaluates whether a given oxidative-stress profile is more compatible with cancer or control status at the time of sampling, and does not quantify individual risk of developing breast cancer in the future. Prospective studies with baseline biomarker assessment and long-term follow-up, such as the classic endocrine-based investigations led by Bulbrook et al. would be required to determine whether similar models can genuinely predict incident breast cancer in asymptomatic populations and support primary prevention strategies [80,81].

However, while its ability to classify breast cancer versus control status is robust, the model shows significant limitations in accurately diagnosing SLN metastatic spread, a challenge that reflects the inherent complexity and heterogeneity of metastasis [47,82]. Metastasis is a multi-step process involving numerous genetic and epigenetic alterations, as well as interactions with the tumor microenvironment [14,15], making it a difficult target for predictive models. Metastatic heterogeneity arises from complex evolutionary processes, with multiclonal metastasis involving genetically distinct tumor clones that can respond differently to therapeutic interventions [83,84,85]. The biomarkers used in our study may not fully capture the complexity of this process, which could explain the limited performance in diagnosing SLN metastatic disease [18,84,86].

When comparing our findings with prior AI and machine learning studies in oncology, it is clear that numerous investigations have successfully employed these techniques to enhance diagnostic accuracy in cancer [42,44]. Machine learning algorithms have been used to analyze various types of data, including imaging data, genomic data, and clinical data, to improve cancer diagnosis, prognosis, and treatment planning [39,65]. AI models can achieve AUC values ranging from 0.779 to 0.862 in cancer prediction, with multi-layer perceptron classifiers showing superior performance [87]. Our work builds on this foundation by incorporating oxidative stress biomarkers, a choice supported by extensive evidence linking these biomarkers to cellular damage and early carcinogenic events [88]. Oxidative stress, resulting from an imbalance between the production of reactive oxygen species and the body’s antioxidant defenses, has been implicated in various stages of cancer development, including initiation, promotion, and progression [31,40,79,86,89,90].

The high accuracy of our model in distinguishing women with breast cancer from healthy controls is consistent with its effective identification of biochemical signals, such as alterations in lipid peroxidation and antioxidant enzyme activities, which are critical indicators of oxidative stress [25,34,66,91]. Lipid peroxidation, a marker of oxidative damage to cell membranes, and changes in the activity of antioxidant enzymes, such as superoxide dismutase and glutathione peroxidase, reflect the cellular response to oxidative stress [32,37,38]. Breast cancer patients exhibit significantly lower total antioxidant capacity and higher oxidized LDL levels compared to healthy controls, with specific oxidative stress-related genes showing close interactions with tumor immune cells and the tumor microenvironment [36,92,93]. For breast cancer classification, the few misclassified cases corresponded to individuals whose oxidative-stress profiles lay close to the decision thresholds or exhibited intermediate biomarker levels overlapping between cancer and control groups. In contrast, misclassifications in SLN status were more frequent, particularly between macrometastasis and control or between macro- and micrometastasis, which we attribute both to the limited sample size and to the partial overlap of systemic oxidative signatures across metastatic categories. Together, these findings support the view that oxidative stress alone is insufficient to achieve highly precise SLN stratification in this cohort. Nonetheless, the challenge of accurately diagnosing metastasis persists, likely due to the dynamic and multifactorial nature of metastatic processes [94].

The rationale for selecting oxidative stress biomarkers is rooted in their established association with both cellular damage and tumor initiation, making them ideal candidates for models aimed at non-invasive disease detection and risk stratification [67]. Oxidative stress can induce DNA damage, protein modifications, and lipid peroxidation, all of which can contribute to the development of cancer [28,37,38]. ROS-induced DNA damage includes base modifications such as 8-hydroxydeoxyguanosine (8-OHdG), strand breaks, and cross-linking, which can lead to mutations and genomic instability if not adequately repaired [89]. However, the dual role of oxidative stress, driving carcinogenesis on one hand and contributing less directly to metastasis on the other, may partly explain the model’s differential performance across these two clinical endpoints. While oxidative stress can promote tumor growth and angiogenesis, its role in the later stages of metastasis, such as invasion and colonization, is less clear [66]. This duality reinforces the need to further elucidate the underlying biological mechanisms and possibly incorporate additional markers that capture the complexity of metastatic progression. Future studies should explore the integration of biomarkers related to epithelial–mesenchymal transition (EMT), immune response, and angiogenesis to improve the prediction of metastasis [95,96,97,98,99].

The architecture of our machine learning framework is based on an ensemble of decision trees (Random Forest), which was validated through a rigorous leave-one-out cross-validation protocol similar in spirit to those described in earlier studies [42,44]. Deep learning models have demonstrated remarkable performance in various applications, including image recognition, natural language processing, and medical diagnosis [46]. Hybrid deep learning models integrating multiple data types can achieve superior diagnostic accuracy, with some frameworks reporting 93.97% accuracy for histopathological images and 89.87% for ultrasound datasets; these approaches are complementary to, rather than overlapping with, the interpretable tree-based strategy used in our study [100,101,102].

While the integration of heterogeneous data types is a notable strength of many AI approach, the so-called “black box” nature of deep learning poses challenges for clinical interpretability, a concern that has been addressed in the literature through the development of explainable AI methods [103]. XAI techniques aim to provide insights into the decision-making process of AI models, making them more transparent and trustworthy [54]. XAI methods such as SHapley Additive exPlanations (SHAP) and local interpretable model-agnostic explanations (LIME) can enhance interpretability while maintaining high diagnostic accuracy, with some explainable models achieving comparable performance to traditional black-box systems [40,65,78,90,104,105,106]. While the decision trees generated by our Random Forest provide direct, interpretable rules (see Table 3, Table 4, Table 5, Table 6, Table 7 and Table 8), future work should integrate formal XAI methods (SHAP, LIME) to quantify feature contributions to enhance clinical interpretability and practice.

Clinically, the high classification performance of our model in this retrospective cohort suggests potential for earlier identification of women with breast cancer when applied in appropriate clinical scenarios, provided that its performance is confirmed in prospective studies. Such validation could ultimately contribute to more timely interventions and reduced mortality [59]. Early detection and intervention have been shown to improve survival rates and quality of life for cancer patients [4]. Moreover, integrating such classification tools into routine clinical practice is in line with the broader shift toward personalized medicine, where treatment strategies are tailored to the individual patient’s risk profile [22,39,60,64,107]. Personalized medicine aims to provide the right treatment to the right patient at the right time, based on their individual characteristics and risk factors [63]. Consistent with current clinical practice, any potential application of SLN-related predictions would most plausibly be restricted to carefully selected scenarios such as patients receiving neoadjuvant systemic therapy or research settings aimed at refining axillary risk stratification, rather than routine preoperative assessment in the general breast cancer population. The integration of AI-based decision-support models into clinical practice has the potential to revolutionize cancer care and improve patient outcomes [24,60,64,90,108].

Despite these promising aspects, our study is not without limitations. The SLN metastasis analysis was based on a relatively small number of metastasis cases, limiting statistical power for rare events and subgroup analyses. Additionally, no external validation cohort was available, so these results should be interpreted as exploratory. Other issues such as variability in biomarker expression across diverse populations, and potential selection biases are acknowledged and echo concerns raised in previous epidemiological studies [2]. The limited sample size may affect the generalizability of our findings, and the variability in biomarker expression across different populations highlights the need for validation in diverse cohorts. In particular, the analysis of SLN metastasis was based on a relatively small number of metastasis cases, and no external validation cohort was available, so these results should be interpreted as exploratory. Studies have emphasized the importance of diverse datasets for improving the generalizability and fairness of AI models, particularly noting that dataset diversity remains a significant limitation in current XAI applications [109]. In light of these limitations, future research should aim to expand the panel of biomarkers, incorporate longitudinal data to better capture temporal dynamics, and explore hybrid modeling approaches that combine classical statistical techniques with modern AI algorithms to enhance the detection of metastasis [110]. Longitudinal data, which tracks changes in biomarker levels over time, could provide valuable insights into the dynamics of cancer development and progression [11]. Longitudinal multi-omics studies have demonstrated their power in unraveling tumor evolution and therapy response, with temporal profiling revealing molecular progression patterns that precede radiographic evidence of disease progression. ctDNA levels rise prior to radiographic progression in most patients with metastatic disease, and that specific mutations show differential progression rates associated with treatment outcomes [106,111,112,113,114].

## 5. Conclusions

Our findings underscore the model’s robust performance in classifying the presence of breast cancer in a retrospective cohort while highlighting significant gaps in its ability to diagnose SLN metastatic disease. This balanced perspective not only reflects the current state of AI-driven diagnostic tools in oncology but also emphasizes the critical need for continued research. Further refinement of the model by incorporating additional biomarkers, longitudinal data, and explainable AI techniques could enhance its clinical utility and facilitate its adoption in clinical practice. Advancing these models further may help to better integrate non-invasive biomarker information with existing screening and staging strategies, ultimately contributing to improved patient outcomes and the evolution of precision oncology. The ultimate goal is to develop AI-based tools that can accurately assess cancer risk, support early detection efforts, and guide treatment decisions, leading to improved survival rates and quality of life for cancer patients.

## Figures and Tables

**Table 1:** Overall results for both models.

	Accuracy	Precision	Recall	F-Score
Breast cancer classification	0.996	0.994	0.998	0.996
SLN Metastasis classification	0.837	0.842	0.837	0.839

**Table 2:** Confusion matrix for the Random Forest model in classifying breast cancer versus controls. Normalized percentages are included in parentheses.

Class/Prediction	Breast Cancer	Control
Breast cancer	188 (1.0)	0 (0.0)
Control	1 (0.013)	77 (0.987)

**Table 3:** Decision rules corresponding to the terminal nodes of the first tree generated by the Random Forest model. The row summarizes the conditions leading to a specific predicted diagnosis, together with the precision and recall achieved by each individual rule.

				Precision	Recall
IF	SOD < 17.8	THEN	diagnosis = Cancer	0.994	0.703
		ELSE	diagnosis = Control	0.961	0.282

**Table 4:** Decision rules corresponding to the terminal nodes of the second tree generated by the Random Forest model. Each row summarizes the conditions leading to a specific predicted diagnosis, together with the precision and recall achieved by each individual rule.

				Precision	Recall
IF	CAT < 9.75 AND Carbonyl < 0.33	THEN	diagnosis = Cancer	0.978	0.334
IF	CAT < 9.75 AND Carbonyl ≥ 0.33 AND age < 47	THEN	diagnosis = Control	1.000	0.011
IF	CAT < 9.75 AND Carbonyl ≥ 0.33 AND age ≥ 47	THEN	diagnosis = Cancer	0.923	0.045
IF	CAT > 9.75 AND GPx < 71.61	THEN	diagnosis = cancer	0.972	0.271
IF	CAT > 9.75 AND GPx > 71.61	THEN	diagnosis = Control	0.922	0.338

**Table 5:** Decision rules corresponding to the terminal nodes of the third tree generated by the Random Forest model. Each row summarizes the conditions leading to a specific predicted diagnosis, together with the precision and recall achieved by each individual rule.

				Precision	Recall
IF	GPx < 71.77 AND SOD < 17.73	THEN	diagnosis = Cancer	0.973	0.687
IF	GPx < 71.77 AND SOD > 17.73	THEN	diagnosis = Control	1.000	0.004
IF	GPx ≥ 71.77	THEN	diagnosis = Control	0.936	0.293

**Table 6:** Decision rules corresponding to the terminal nodes of the fourth tree generated by the Random Forest model. Each row summarizes the conditions leading to a specific predicted diagnosis, together with the precision and recall achieved by each individual rule.

				Precision	Recall
IF	GPx < 71.61 AND SOD < 17.73	THEN	diagnosis = Cancer	0.962	0.680
IF	GPx < 71.61 AND SOD > 17.73	THEN	diagnosis = Control	1.000	0.004
IF	GPx ≥ 71.61	THEN	diagnosis = Control	0.910	0.293

**Table 7:** Decision rules corresponding to the terminal nodes of the fifth tree generated by the Random Forest model. Each row summarizes the conditions leading to a specific predicted diagnosis, together with the precision and recall achieved by each individual rule.

				Precision	Recall
IF	BMI < 24.45 AND TAC < 52.42 AND tGSH < 4.03	THEN	diagnosis = Cancer	0.941	0.064
IF	BMI < 24.45 AND TAC < 52.42 AND tGSH ≥ 4.03	THEN	diagnosis = Control	0.800	0.015
IF	BMI < 24.45 AND TAC ≥ 52.42 AND CAT < 9.48	THEN	diagnosis = Cancer	0.667	0.008
IF	BMI < 24.45 AND TAC ≥ 52.42 AND CAT ≥ 9.48	THEN	diagnosis = Control	0.957	0.083
IF	BMI ≥ 24.45 AND BMI < 25.13	THEN	diagnosis = Control	0.925	0.094
IF	BMI ≥ 25.13 AND tGSH < 4.13	THEN	diagnosis = Cancer	0.943	0.624
IF	BMI ≥ 25.13 AND tGSH ≥ 4.13 AND GPx < 71.79	THEN	diagnosis = Cancer	1.000	0.023
IF	BMI ≥ 25.13 AND tGSH ≥ 4.13 AND GPx ≥ 71.79	THEN	diagnosis = Control	1.000	0.086

**Table 8:** Decision rules corresponding to the terminal nodes of the sixth tree generated by the Random Forest model. Each row summarizes the conditions leading to a specific predicted diagnosis, together with the precision and recall achieved by each individual rule.

				Precision	Recall
IF	TAC < 47.92 AND GPx < 71.37	THEN	diagnosis = Cancer	0.982	0.417
IF	TAC < 47.92 AND GPx ≥ 71.37	THEN	diagnosis = Control	1.000	0.008
IF	TAC ≥ 47.92 AND status = Postmenopause AND tGSH < 3.97	THEN	diagnosis = Cancer	0.982	0.207
IF	TAC ≥ 47.92 AND status = Postmenopause AND tGSH ≥ 3.97	THEN	diagnosis = Control	1.000	0.053
IF	TAC ≥ 47.92 AND status = Premenopause AND SOD < 19.02	THEN	diagnosis = Cancer	0.962	0.098
IF	TAC ≥ 47.92 AND status = Premenopause AND SOD ≥ 19.02	THEN	diagnosis = Control	0.983	0.214

**Table 9:** Confusion matrix for the Random Forest model in classifying SLN metastases. Normalized percentages are included in parentheses.

Class/Prediction	Macrometastasis	Micrometastasis	Control
Macrometastasis	49 (0.860)	2 (0.035)	6 (0.105)
Micrometastasis	2 (0.035)	54 (0.947)	1 (0.018)
Control	10 (0.175)	4 (0.070)	43 (0.754)

## Data Availability

The datasets presented in this article are not readily available because privacy or ethical restrictions.

## Abbreviations

The following abbreviations are used in this manuscript:

8-OHdG	8-hydroxydeoxyguanosine
AI	Artificial intelligence
AUC	Area under the receiver operating characteristic curve
BMI	Body mass index
CAT	Catalase
CK19	Cytokeratin 19
CNN	Convolutional neural networks
ctDNA	Circulating tumor DNA
EMT	Epithelial–mesenchymal transition
GPx	Glutathione peroxidase
LDL	Low-density lipoprotein
LIME	Local interpretable model-agnostic explanations
OSNA	One-step nucleic acid amplification
ROS	Reactive oxygen species
SHAP	SHapley Additive exPlanations
SLN	Sentinel lymph node
SMOTE	Synthetic minority over-sampling technique
SOD	Superoxide dismutase
TAC	Total antioxidant capacity
tGSH	Total glutathione
XAI	Explainable artificial intelligence
